# Sex-based association between high-density lipoprotein cholesterol and adverse outcomes after coronary artery bypass grafting

**DOI:** 10.1186/s12872-024-03806-1

**Published:** 2024-04-05

**Authors:** Sara Montazeri Namin, Ali Moradi, Hamed Tavolinejad, Ali Vasheghani-Farahani, Arash Jalali, Mina Pashang, Saeed Sadeghian, Jamshid Bagheri, Soheil Mansourian, Mehdi Mehrani, Kaveh Hosseini, Sina Rashedi, Masih Tajdini

**Affiliations:** 1 Tehran Heart Center, Cardiovascular Diseases Research Institute, Tehran University of Medical Sciences, Tehran, Iran; 2 Cardiac Primary Prevention Research Center, Cardiovascular Diseases Research Institute, Tehran University of Medical Sciences, Tehran, Iran

**Keywords:** Coronary artery bypass graft surgery, High-density lipoprotein cholesterol, Major adverse cardiovascular and cerebrovascular events, Mortality

## Abstract

**Background:**

High-density lipoprotein cholesterol (HDL-C) is shown to be an independent protective factor against coronary artery diseases (CAD). Yet there are limited studies focusing on the association between HDL-C and coronary artery bypass graft (CABG) surgery outcomes.

**Hypothesis:**

Low levels of HDL-C are associated with higher incidence of adverse outcomes in patients undergoing CABG.

**Methods:**

This registry-based study included 17,772 patients who underwent elective isolated CABG between 2007 and 2017. Patients were classified into low and desirable HDL-C groups based on their serum HDL-C levels at admission and were followed for one-year post-surgery. The study population included 13,321 patients with low HDL-C and 4,451 with desirable HDL-C. proportional hazard Cox models were performed to evaluate the association between HDL-C levels and incidence of mortality as well as major adverse cardiovascular and cerebrovascular events (MACCE), while adjusting for potential confounders. Moreover, participants were stratified based on sex and the association was also investigated in each subgroup separately.

**Results:**

No significant difference was found between the groups regarding incidence of both mortality and MACCE, after adjusting with Inverse Probability Weighting (IPW) [HR (95%CI): 0.84 (0.46–1.53), p-value:0.575 and HR (95% CI): 0.91 (0.56–1.50), p-value:0.733, respectively]. According to the sex-based subgroup analysis, no significant association was observed after adjustment with IPW analysis. However, as we examined the association between the interaction of HDL-C levels, sex and cardiovascular outcomes, we found a significant association (HR;1.19 (95%CI: 1.04–1.45); *p* = 0.030).

**Conclusion:**

HDL-C level was not associated with either mortality or MACCE during one year after CABG procedure. Sex-based analysis showed that in males, HDL-C is significantly more protective against these outcomes, compared to females. Further studies are necessary to elucidate the exact mechanisms mediating such association.

**Supplementary Information:**

The online version contains supplementary material available at 10.1186/s12872-024-03806-1.

## Introduction

Dyslipidemia is associated with the development of atherosclerotic cardiovascular diseases, a leading cause of morbidity and mortality worldwide [1]. As revealed by several epidemiological studies, high-density lipoprotein cholesterol (HDL-C) plays an independent protective role in coronary artery disease (CAD) pathogenesis [2]. According to the previous experimental studies, the atheroprotective effects of HDL-C are probably due to reversing macrophage cholesterol transport, as well as its anti-inflammatory, antioxidant, and antithrombotic properties [3–5].

Even though several studies have pointed out the role of HDL-C level in the prediction of mortality and cardiovascular events in patients with CAD [6, 7], less is known about the association between HDL-C levels and long-term adverse outcomes after CABG and the literature remains controversial in this respect [8–10]. Meanwhile, in patients undergoing percutaneous coronary intervention (PCI), low HDL-C level is suggested to be associated with revascularization, peri-procedural acute myocardial infarction (MI), and 1-year mortality [11–13]. This gave rise to the perspective that higher serum level of HDL-C might be accompanied by better outcomes following coronary artery bypass graft (CABG).

In addition, sex-based disparities in lipid metabolism have long been recognized, with accumulating evidence suggesting variations in HDL-C levels between males and females [14]. Understanding these sex differences is crucial, as it may impact the preoperative assessment and management of patients undergoing CABG [15, 16].

In the present study, we evaluated the association between baseline level of HDL-C and post-CABG outcomes in a large retrospective cohort of patients with CAD who underwent elective isolated CABG. Moreover, we stratified the sample population based on their sex and investigated this association in the subgroups as well. To the best of our knowledge, this is the first study determining the sex-specific association between HDL-C and post-CABG outcomes.

## Method

This registry-based cohort study was performed at Tehran Heart Center (THC) on patients who underwent surgery between January 1, 2007 to December 31, 2017. According to the standard hospital database policy, baseline clinical, demographic, procedural, and discharge information were gathered along with 1-year outcomes. The detailed registry protocol has been reported elsewhere [17]. This study was approved by the institutional review board and the research ethics committee at Tehran Heart Center (Approval ID: IR.TUMS.THC.REC.1400.045).

### Population

All patients who underwent elective isolated CABG during the study period and were discharged without experiencing in-hospital post-surgical acute myocardial infarction or stroke were included in the analysis. Our exclusion criteria were as follows: urgent CABG, concurrent valve surgery, in-hospital MI, stroke or death, and absence of patient data.

### Exposure

HDL-C level was measured during index hospitalization for surgery. The population was stratified based on serum HDL-C levels into low and desirable HDL-C groups. Low HDL-C levels were defined as < 40 mg/dl in males and < 50 mg/dl in females [18].

### Patient follow-up

While enrolled in the study, patients were followed by intermittent clinic visits at 1, 6, and 12 months after surgery. As well, telephone home assessments at 3 and 9 months were done. All patients were treated based on current guidelines including smoking cessation counseling, aggressive diabetes management, aspirin administration, beta-blockers, angiotensin converting enzyme inhibitors, and statin medications [19]. Patients received the standard care concerning post-surgery lipid therapy. If no contraindication existed, cardiologists were advised to administer statin therapy postoperatively to each patient included in the study as recommended in the current guidelines [19]. The statin type and dose were left to the prescribing physician’s discretion and not dictated by the study protocol.

Serum level of HDL-C was measured prior to CABG surgery. Office or telephone follow-ups were performed every 3 months to ensure medication compliance, including statin therapy. Twelve-month clinical follow-up was done for all patients.

### Study outcomes and definitions

Outcomes of interest were all-cause death and major adverse cardiovascular and cerebrovascular events (MACCE), including death, acute coronary syndrome, stroke, transient ischemic attack (TIA), and repeat revascularization. In a subgroup analysis, the study outcomes were evaluated separately among male and female participants. Acute coronary syndrome was defined as a clinical presentation of acute myocardial ischemia (unstable angina) or infarction (ST-segment elevation myocardial infarction or non-ST segment elevation myocardial infarction) [20]. Stroke was defined as a disturbance of cerebral function, lasting more than 24 h or leading to death, with no apparent cause other than of vascular origin. TIA was determined as a transient episode of neurological dysfunction following focal brain, spinal cord, or retinal ischemia, without acute infarction [21]. Repeat revascularization was defined as unplanned repeat revascularization of a coronary artery with PCI or CABG [22].

### Study analysis

Normal distribution among quantitative data was assessed based on histogram drawing, central tendency and dispersion indices, and Smirnov Kolmogorov statistical test. Data with normal distribution were reported as mean ± SD, otherwise, it was reported as mediocre (25 percentile-75 percentile). Quantitative data among the two groups were compared by the Independent Sample t-test or Mann-Whitney test. Qualitative data were reported as percentages and were assessed using the Chi-square test.

Outcomes were analyzed using the proportional Hazard Cox model and the relation between HDL-C level and outcomes were reported as Hazard ratio (HR) and 95% confidence interval (95% CI). To account for potential confounders in results, we used inverse probability weighting (IPW) analysis in which the probability of patients being in either of the exposure groups was assessed by confounder variables including age, history of diabetes, hypertension, heart attack, heart failure, coronary revascularization, chronic kidney disease, chronic pulmonary disease, history of opioids use, family history of heart disease, surgery without pump, number of vascular grafts, BMI, creatinine level, statin therapy, LDL-C and triglyceride levels. In addition, we examined the association between HDL-C levels × sex interaction (as predictor) and cardiovascular outcomes, including mortality and MACCE. The multiplicative interaction term was assessed in the Cox model to measure effect on a hazard ratio (relative) scale. Moreover, the χ^2^ test of the correlation between scaled Schoenfeld residuals and log-transformed time was checked to confirm the proportional hazards assumption.

HR plots were created by the “plotHR” function, which is a generic function that employs the “predict” function on the regression model after specifying a penalized spline basis for the predictor using the “pspline” argument. The HR plots visualize the smooth relationship between a variable and the hazard ratio. The grey histogram on the bottom displays the distribution of the predictor (HDL-C). In addition, we incorporated the impact of HDL-C on the specific components of MACCE separately in a supplement Table 3.

R (version 4.0.2) software and survival and survminer packages were utilized for statistical analysis. The incidence rate per 1000 person-years was assessed based on the duration between surgery and the first outcome or last follow-up. P-values lower than 0.05 were considered statistical significance.

## Results

### Baseline characteristics

**Fig. 1:**
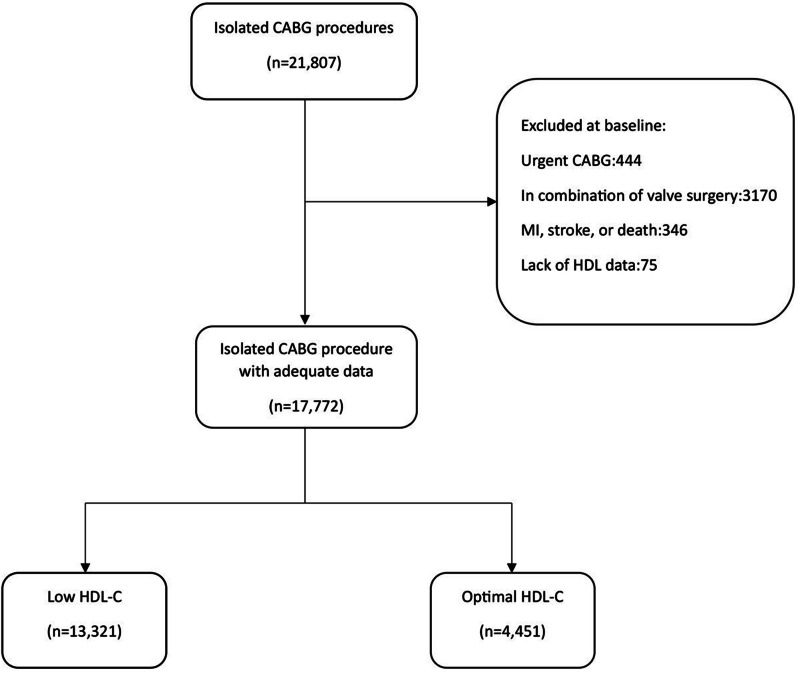
Flow chart of the study cohort

Among 21,807 eligible patients, 444 patients went through urgent CABG, and 3170 patients had a concurrent operation on heart valves. Moreover, 346 patients experienced MI, stroke, or death before being discharged and therefore, were excluded. Among 17,847 patients, data for baseline HDL-C was not available in 75 cases. The final study population included 17,772 patients, including 13,321 patients (75%) with‌ low HDL-C (the exposed population), and 4,451 patients (25%) with desirable HDL-C (Fig. 1).

Among the two groups of desirable and low HDL-C, patients with low HDL-C were lower in age (66.7 ± 9.7 vs. 68.8 ± 9.5; *p* < 0.001). Furthermore, women formed a higher proportion of the low HDL-C group (29.8% vs. 15.9%; *p* < 0.001). Evaluation of cardiac risk factors demonstrates that patients with low HDL-C levels compared to the desirable HDL-C level group were more involved with diabetes (42.7% vs. 31.1%; *p* < 0.001) and hypertension (55.0% vs. 49.3%; *p* < 0.001). Moreover, patients with low HDL-C levels are more involved with chronic kidney diseases (2.2% vs. 1.5%; *p* = 0.002). On the other hand, patients with low HDL-C levels reported lower opium use (15.9% vs. 17.9%; *p* < 0.001). Concerning body mass index (BMI), although statistical differences between the two groups were significant, the average BMI of the two groups was too close which may not suggest any meaningful clinical difference (27.5 ± 4.2 vs. 26.4 ± 4.0 kg/m^2^; *p* < 0.001). Off pump coronary artery bypass grafting were performed in 1685 (9.6%) patients in which the low HDL-C group formed a higher proportion (9.9% vs. 8.7%; *p* = 0.018).

Lab data analysis shows that the low HDL-C group had lower LDL-C values (94.4 ± 36.6 vs. 102.5 ± 39.2 mg/dL; *p* < 0.001) and higher TG levels (median of 140 vs. 110 mg/dL; *p* < 0.001). Table 1 demonstrates the demographic, clinical, and procedural characteristics of the general population.

**Table 1:** , baseline characteristics of patients at the time of surgery

Variables	Total (*n* = 17,772)	Low HDL-C (*n* = 13,321)	Desirable HDL-C (*n* = 4,451)	P value
Mean, mg/dL	36.8 ± 6.1	32.9 ± 5.6	48.7 ± 7.7	**< 0.001**
Age, years	67.2 ± 9.7	66.7 ± 9.7	68.8 ± 9.5	**< 0.001**
Female sex	4674 (26.3%)	3968 (29.8%)	706 (15.9%)	**< 0.001**
Diabetes mellitus	7063 (39.8%)	5680 (42.7%)	1383 (31.1%)	**< 0.001**
Hypertension	9515 (53.5%)	7320 (55.0%)	2195 (49.3%)	**< 0.001**
Current smoking	3155 (17.8%)	2375 (17.9%)	780 (17.6%)	0.661
Opium use	2894 (16.4%)	2101 (15.9%)	793 (17.9%)	**0.001**
FHpCVD	6501 (36.6%)	4911 (36.9%)	1590 (35.7%)	0.170
BMI, kg/m2	27.2 ± 4.2	27.5 ± 4.2	26.4 ± 4.0	**< 0.001**
Previous MI	5965 (33.6%)	4495 (33.7%)	1470 (33%)	0.385
Previous HF	490 (2.8%)	378 (2.9%)	112 (2.6%)	0.254
CKD	362 (2%)	297 (2.2%)	65 (1.5%)	**0.002**
COPD	626 (3.5%)	461 (3.5%)	165 (3.7%)	0.446
Previous CVA	1177 (6.7%)	882 (6.7%)	295 (6.7%)	0.984
PAD	323 (1.8%)	238 (1.8%)	85 (1.9%)	0.596
Previous CABG	88 (0.5%)	62 (0.5%)	26 (0.6%)	0.329
Previous PCI	1337 (7.5%)	997 (7.5%)	340 (7.6%)	0.735
LDL-C, mg/dL	96.4 ± 37.4	94.4 ± 36.6	102.5 ± 39.2	**< 0.001**
Triglycerides, mg/dL	132 (98–180)	140 (105–190)	110 (82–148)	**< 0.001**
EF, %	50 (40–55)	48 (40–55)	50 (40–55)	**< 0.001**
Number of grafts				0.446
1	338 (1.9%)	261 (2%)	77 (1.7%)	
2	1995 (11.2%)	1486 (11.2%)	509 (11.4%)	
3	6805 (38.3%)	5141 (38.6%)	1664 (37.4%)	
4	7041 (39.6%)	5254 (39.5%)	1787 (40.2%)	
5+	1581 (8.9%)	1169 (8.8%)	412 (9.3%)	
OPCAB	1685 (9.6%)	1303 (9.9%)	382 (8.7%)	**0.018**
ACEi or ARB	11,896 (69.1%)	8970 (69.3%)	2926 (68.2%)	0.179
Statin	15,600 (89.7%)	11,719 (89.7%)	3881 (89.7%)	0.910
Aspirin	12,413 (72.1%)	9319 (72%)	3094 (72.2%)	0.856
Beta Blockers	14,354 (82.2%)	10,802 (82.4%)	3552 (81.7%)	0.335

HDL-C, high density lipoprotein cholesterol; FHpCVD, family history of cardiovascular disease; BMI, body mass index; MI, myocardial infarction; HF, heart failure; CKD, chronic kidney disease; COPD, chronic obstructive pulmonary disease; CVA, cerebrovascular accident; PAD, peripheral arterial disease; CABG, coronary artery bypass grafting; PCI, percutaneous coronary intervention; LDL-C, low density lipoprotein cholesterol; EF, ejection fraction; OPCAB, off-pump coronary artery bypass; ACEi, Angiotensin-converting-enzyme inhibitors; ARB, Angiotensin receptor blockers

### Association between HDL-C levels and outcomes

**Fig. 2:**
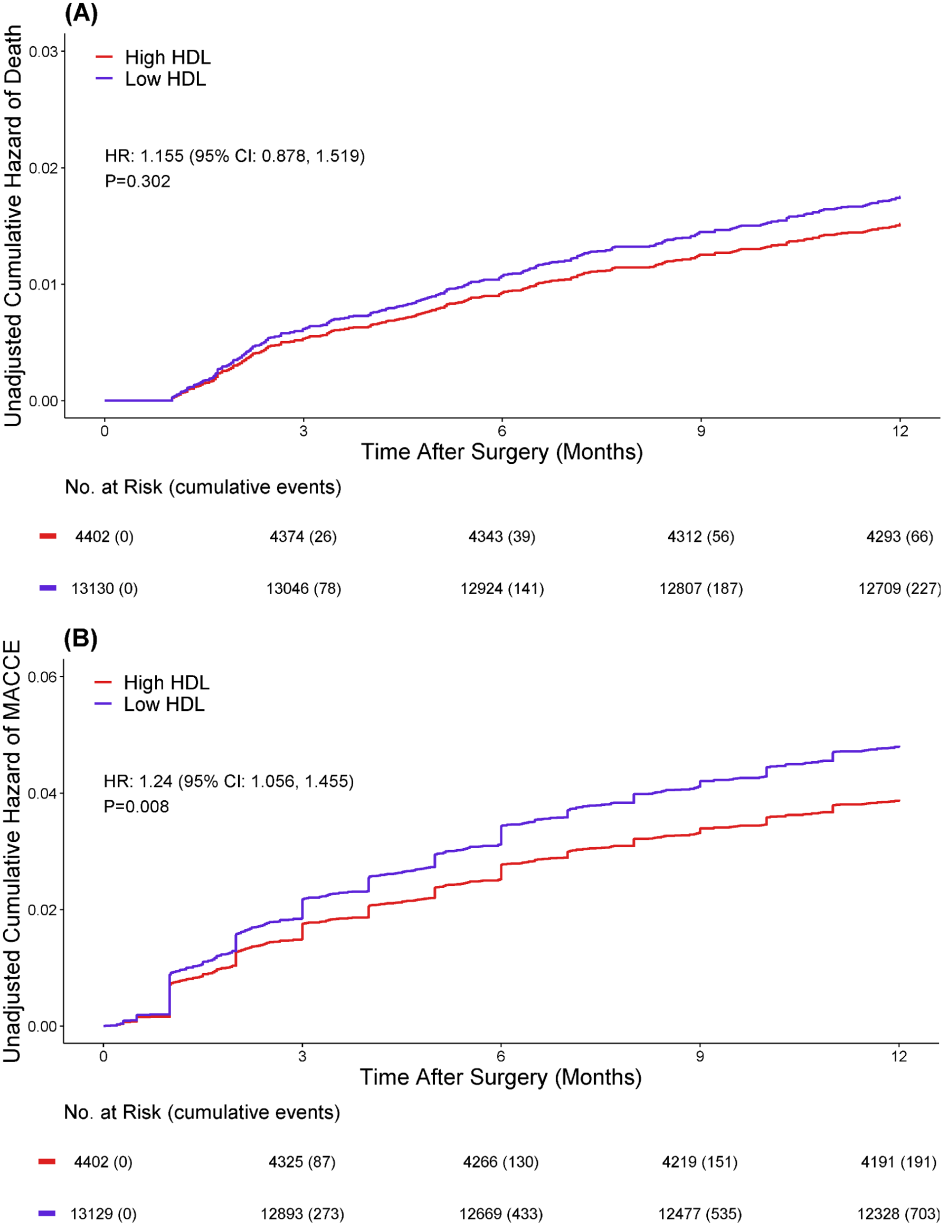
Survival curve for (A) 1-year post surgery mortality; and (B) MACCE outcomes (unadjusted)

In relation to the 1-year post-surgery mortality across both groups, an HR of 1.15 means no significant difference for risk of death during the follow-up period (HR: 1.15; 95% CI = 0.88–1.52; *p* = 0.302; Refer to Table 2; Fig. 2.A). However, concerning 1-year post-surgery MACCE composed outcomes, the low HDL-C group experienced more adverse results (HR: 1.24; 95%CI: 1.06–1.45; *p* = 0.008; Table 2. Figure 2.B).

**Table 2:** One-year post surgery outcomes

Outcomes	No. of events	Incidence rate per 1000 person-years (95% CI)	HR (95% CI)	P value
Incident death (Crude)				
Desirable HDL-C	66/4451	15.19 (11.75–19.32)	Reference	0.302
Low HDL-C	227/13,321	17.56 (15.35–19.99)	1.15 (0.88–1.52)	
Incident death (IPW-adjusted)				
Desirable HDL-C	66/4451	15.19 (11.75–19.32)	Reference	0.575
Low HDL-C	227/13,321	17.56 (15.35–19.99)	0.84 (0.46–1.54)	
Incident MACCE (Crude)				
Desirable HDL-C	191/4451	44.70 (38.59–51.51)	Reference	0.008
Low HDL-C	704/13,321	55.51(51.49–59.77)	1.24 (1.06–1.45)	
Incident MACCE (IPW-adjusted)				
Desirable HDL-C	191/4451	44.70 (38.59–51.51)	Reference	0.733
Low HDL-C	704/13,321	55.51(51.49–59.77)	0.92 (0.56–1.50)	

HR, hazard ratio; CI, confidence interval; HDL-C, high density lipoprotein cholesterol; MACCE, major adverse cardiovascular and cerebrovascular events

**Fig. 3:**
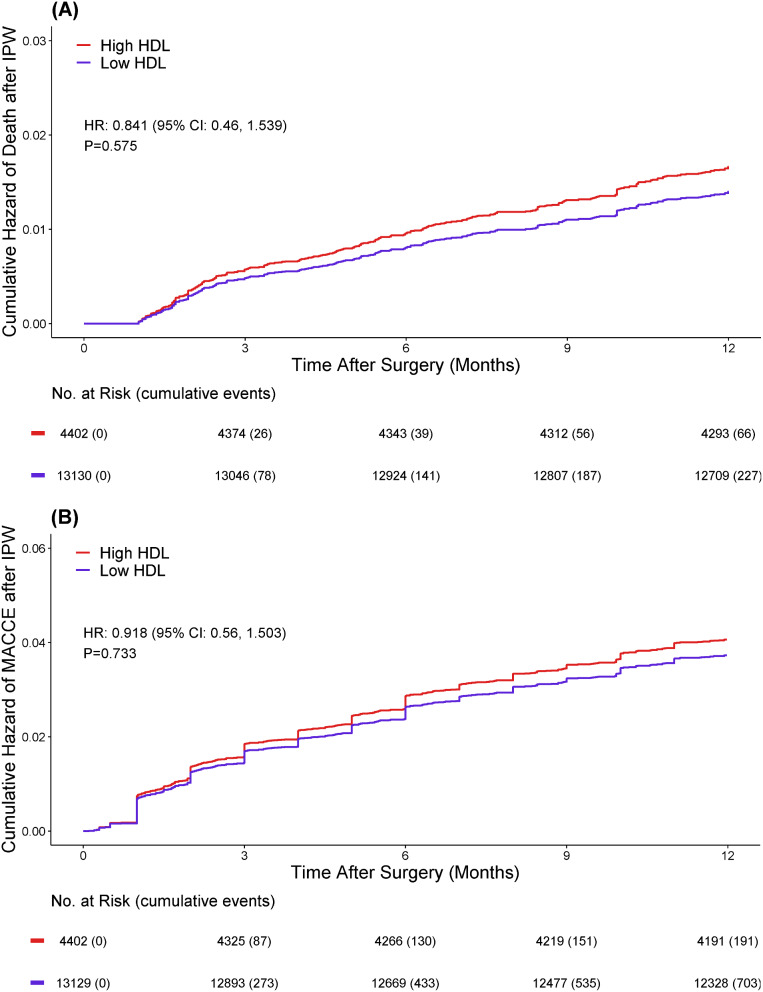
Survival curve for (A) 1-year post surgery mortality; and (B) MACCE outcomes, after IPW analysis

IPW analysis of 1-year post-surgery mortality revealed no association between HDL-C level and death (HR: 0.84; 95%CI :0.46–1.54; *p* = 0.575; Fig. 3.A). Regarding MACCE, after IPW analysis, the observed association lost its statistical significance (HR: 0.92; 95% CI: 0.56–1.50; *p* = 0.733; Fig. 3.B).

**Fig. 4:**
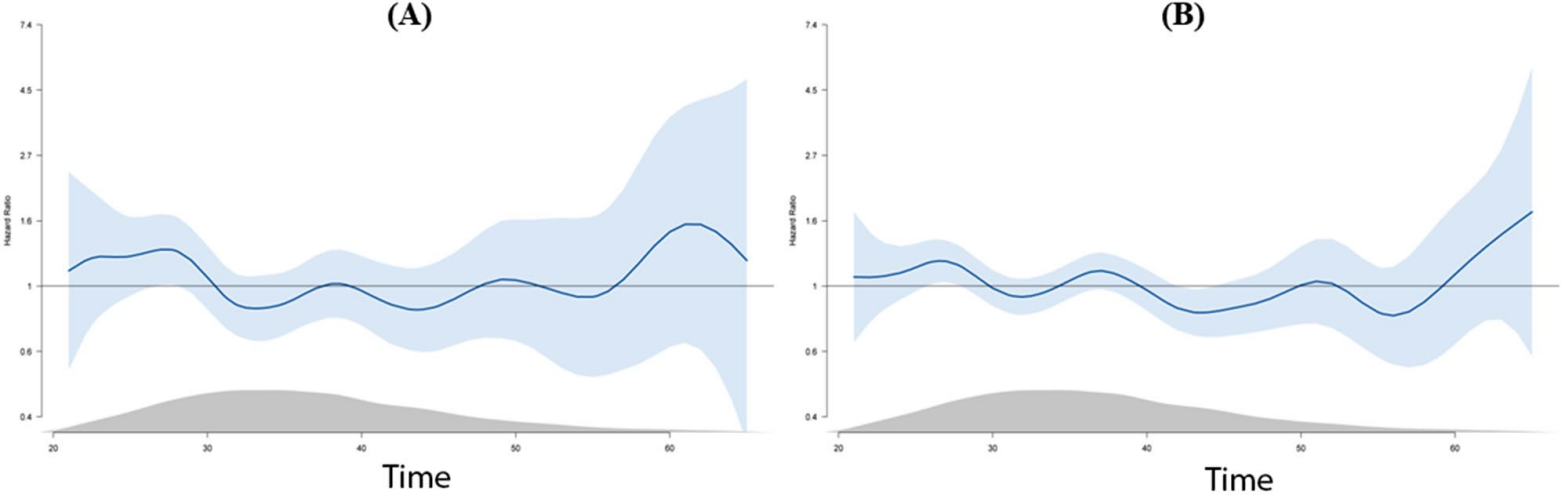
Plots for hazard ratio (HR) with 95% confidence interval in different levels of HDL-C for (A) mortality; (B) MACCE; gray shaded area shows the HDL-C levels distribution

In addition, hazard plots did not reveal an inverse and uniform relation between HDL-C levels and incidence rate of mortality, as well as MACCE (Fig. 4).

### Sex-specific association between HDL-C levels and outcomes

Because of desirable HDL-C levels and metabolic differences among males and females, the events were studied in the male (13,098) and female (4,674) subgroups separately. Among the female subgroup, 1-year post-surgery outcome results analysis demonstrated no association between HDL-C levels and mortality (HR: 0.93; 95%CI: 0.51–1.69; *p* = 0.835) or MACCE (HR: 1.06; 95%CI: 0.75–1.52; *p* = 0.715). After IPW analysis, the same trend was observed between HDL-C levels and mortality (HR: 0.82; 95%CI: 0.36–1.85; *p* = 0.643) or MACCE (HR: 0.90; 95%CI: 0.52–1.56; *p* = 0.727). Furthermore, among the male subgroup, the study of 1-year post-surgery outcomes showed no association between HDL-C level and death (HR: 1.20; 95%CI: 0.88–1.64, *p* = 0.237). On the other hand, there was a significant association between HDL-C level and MACCE (HR: 1.27; 95%CI: 1.06–1.52; *p* = 0.009). Moreover, after IPW analysis there was no significant association between HDL-C levels and mortality (HR: 1.15; 95%CI: 0.70–1.87, *p* = 0.573) or MACCE (HR: 1.22: 95%CI: 0.95–1.55, *p* = 0.109).

Despite the lack of a significant correlation between HDL-C levels and CABG outcomes in the IPW analysis, a significant association was found when we evaluated the association between the interaction of HDL-C levels and sex (HDL-C × sex) and MACCE (1.19; 95% CI: 1.04–1.45); *p* = 0.030), suggesting that higher HDL-C levels provide greater protection among males compared to females.

## Discussion

Our main results in this retrospective registry-based cohort study indicated no significant association between baseline serum level of HDL-C and cardiovascular outcomes—at 1-year followed among patients who had undergone CABG. Although a meaningful link between low HDL-C level and higher MACCE risk was detected in the unadjusted analysis, this association transformed into a non-significant trend after considering potential confounding variables. Moreover, an examination of the interaction between HDL-C levels and sex suggested that HDL-C levels might serve higher grades of protectiveness within the male patients compared to female patients.

HDL-C has been suggested as an independent protective factor for atherosclerotic cardiovascular diseases. Previous studies have demonstrated that low HDL-C level is associated with a greater risk of mortality and cardiovascular events in CAD patients [23, 24]. In particular, several studies have shown that there is a strong association between HDL-C levels and the incidence of post-PCI outcomes in patients undergoing percutaneous coronary intervention (PCI) [25, 26]. However, the literature remains heterogenous in this respect as Izuhara et al. studied 10,391 patients undergoing PCI at baseline in a 5-year cohort and found no significant association between baseline HDL-C level and major adverse cardiovascular events (MACE) [27].

There are heterogenous data concerning the association between HDL-C levels and outcomes following CABG. In a cohort investigation, post-CABG patients were categorized into two groups (502 patients in each group) based on their baseline HDL-C levels. While the baseline characteristics of the groups were comparable, Angeloni et al. observed no difference in the incidence of cardiovascular outcomes between the groups, which is in line with the results of this study [4]. Stratifying the participants based on their sex, we extended the investigation regarding this association and found that male patients with low HDL-C levels compared to females experience worse cardiovascular outcomes following CABG. Foody et al. reported a similar association after studying 432 male patients following CABG showing low HDL-C group (≤ 35 mg/dl) has a higher rate of recurrent MI and revascularization [10]. To address the heterogeneity in the results of related studies, it is necessary to take into account a multitude of factors. Characteristics of the population, including demographics, comorbidities, and genetic elements, play a significant role in the variations observed. Furthermore, the disparities in follow-up durations, sample size, definitions of outcomes, and effect sizes across different studies add another layer of complexity to the interpretation [28, 29]. In the present study, we also evaluated this association in a large group of patients and adjusted potential confounders to address these issues. To the best of our knowledge, this is the first study suggesting the presence of a sex-specific pattern in the association between HDL-C level and incidence of cardiovascular outcomes in post-CABG patients. Even though there are studies suggesting that low HDL-C levels are associated with worse cardiovascular outcomes, none has examined this issue separately in each sex [1, 30–33].

We studied patients undergone elective isolated CABG surgery and not general populations. Therefore, CAD in these patients may overlap with other conditions that interrupt HDL-C functioning (e.g., diabetes mellitus, smoking) [2, 34, 35]. In addition, the impact of HDL-C level on post-CABG outcomes might differ according to the intensity of statin therapy, which is commonly used in the clinical management of patients with CAD. As reflected in several studies, a significant association is evident between HDL-C level and cardiovascular events in non-users and those treated with a low-dose statin, but not in the participants receiving aggressive statin therapy [36, 37]. Therefore, future relevant studies should consider adjusting statin intake as a potential confounding factor.

Several limitations are present in the current study. First, the measurement of HDL-C level was limited to the baseline while longitudinal changes could have made the results more accurate. We assumed that during one-year post-CABG surgery follow-up, HDL-C levels would not change considerably. Second, in this study, several confounding variables were noted; however, the probability of residual confounding still remains. Third, given that the current study is single-center, it is necessary to consider a multi-center design for upcoming studies in this field. Forth, Finally, the retrospective nature of the current study prevents us from determining the causal relationship between HDL-C levels and cardiovascular outcomes, therefore only the association could be examined and reported.

## Conclusion

This registry-based cohort study revealed no association between low HDL-C levels and incidence of MACCE including cardiac arrest, stroke, the necessity of revascularization, and mortality during 1-year post-CABG surgery. However, when we stratify the data by sex, analysis of the association of the interaction of HDL-C and sex with cardiovascular outcomes indicates that higher HDL-C levels provide more protection for male patients compared to females. Further research and clinical trials are necessary to clarify the role of measuring HDL-C level in predicting the prognosis of patients undergoing CABG, particularly those at higher risks of cardiovascular events. Moreover, additional investigations are warranted to also evaluate the sex-specific role of HDL-C level in this regard.

### Electronic supplementary material

Below is the link to the electronic supplementary material.

Supplementary Material 1

## Data Availability

The datasets analyzed during the current study are not publicly available due to potential identification of patients. Furthermore, the data from this study belong to Tehran University of Medical Sciences and are not permitted to be published publicly. However, the data can be obtained from the corresponding author upon a reasonable request.
