# Supplementary material for: Sex-based association between high-density lipoprotein cholesterol and adverse outcomes after coronary artery bypass grafting
Source: BMC Cardiovasc Disord. 2024 Apr 5;24:194. doi: 10.1186/s12872-024-03806-1 (PMC10996185; doi:10.1186/s12872-024-03806-1)
Supplement: Supplementary file 1 — Supplementary Material 1 [file 12872_2024_3806_MOESM1_ESM.docx]

**Sex-based association between high-density lipoprotein cholesterol and adverse outcomes after coronary artery bypass grafting**

**Supplemental Table 1.** Baseline characteristics of Female subgroup

| **Variables** | **Total**  (n = 4,674) | **Desirable HDL-C**  (n = 706) | **Low HDL-C**  (n = 3,968) | **p-value** |
| --- | --- | --- | --- | --- |
| **Age, years** | 68.44 ± 8.99 | 70.31 ± 8.32 | 68.11 ± 9.06 | **<0.001** |
| **Diabetes mellitus** | 2,677 (57.4%) | 336 (47.8%) | 2,341 (59.1%) | **<0.001** |
| **Hypertension** | 3,404 (72.8%) | 495 (70.1%) | 2,909 (73.3%) | 0.078 |
| **Current smoking** | 115 (2.5%) | 10 (1.4%) | 105 (2.7%) | 0.053 |
| **Opium use** | 76 (1.6%) | 6 (0.9%) | 70 (1.8%) | 0.169 |
| **FHpCVD** | 1,986 (42.5%) | 297 (42.1%) | 1,689 (42.6%) | 0.805 |
| **BMI, kg/m2** | 28.80 ± 4.76 | 28.40 ± 4.58 | 28.87 ± 4.78 | **0.013** |
| **Previous MI** | 1,220 (26.1%) | 176 (24.9%) | 1,044 (26.3%) | 0.441 |
| **Previous HF** | 116 (2.5%) | 16 (2.3%) | 100 (2.6%) | 0.671 |
| **CKD** | 93 (2.0%) | 12 (1.7%) | 81 (2.1%) | 0.544 |
| **COPD** | 182 (3.9%) | 36 (5.1%) | 146 (3.7%) | 0.074 |
| **Previous CVA** | 312 (6.8%) | 55 (7.9%) | 257 (6.6%) | 0.199 |
| **PAD** | 85 (1.8%) | 14 (2.0%) | 71 (1.8%) | 0.730 |
| **Previous CABG** | 13 (0.3%) | 2 (0.3%) | 11 (0.3%) | >0.999 |
| **Previous PCI** | 326 (7.0%) | 48 (6.8%) | 278 (7.0%) | 0.842 |
| **LDL-C, mg/Dl** | 104.11 ± 40.20 | 111.40 ± 40.23 | 102.81 ± 40.06 | **<0.001** |
| **Triglycerides, mg/dL** | 163.39 ± 90.24 | 125.78 ± 56.24 | 170.09 ± 93.46 | **<0.001** |
| **Creatinine, mg/dL** | 0.85 ± 0.53 | 0.86 ± 0.63 | 0.85 ± 0.51 | 0.818 |
| **EF, %** | 47.88 ± 8.54 | 49.05 ± 8.09 | 47.67 ± 8.60 | **<0.001** |
| **Number of grafts** |  |  |  | 0.094 |
| **1** | 121 (2.6%) | 17 (2.4%) | 104 (2.6%) |  |
| **2** | 610 (13.1%) | 85 (12.1%) | 525 (13.2%) |  |
| **3** | 1,951 (41.8%) | 315 (44.7%) | 1,636 (41.3%) |  |
| **4** | 1,702 (36.4%) | 234 (33.2%) | 1,468 (37.0%) |  |
| **5** | 287 (6.1%) | 54 (7.7%) | 233 (5.9%) |  |
| **OPCAB** | 449 (9.7%) | 53 (7.6%) | 396 (10.1%) | **0.040** |
| **ACEi** | 1,790 (39.4%) | 276 (40.6%) | 1,514 (39.2%) | 0.487 |
| **Statin** | 4,097 (89.6%) | 611 (89.3%) | 3,486 (89.6%) | 0.807 |
| **Aspirin** | 3,225 (71.1%) | 475 (70.0%) | 2,750 (71.3%) | 0.489 |
| **Beta Blockers** | 3,856 (83.9%) | 551 (80.1%) | 3,305 (84.6%) | **0.003** |

HDL-C, high density lipoprotein cholesterol; FHpCVD, family history of cardiovascular disease; BMI, body mass index; MI, myocardial infarction; HF, heart failure; CKD, chronic kidney disease; COPD, chronic obstructive pulmonary disease; CVA, cerebrovascular accident; PAD, peripheral arterial disease; CABG, coronary artery bypass grafting; PCI, percutaneous coronary intervention; LDL-C, low density lipoprotein cholesterol; EF, ejection fraction; OPCAB, off-pump coronary artery bypass; ACEi, Angiotensin-converting-enzyme inhibitors; ARB, Angiotensin receptor blockers.

**Supplemental Table 2.** Baseline characteristics of Male subgroup

| **Variable** | **Total**  (n =13,098) | **Desirable HDL-C**  (n = 3,745) | **Low HDL-C**  (n = 9,353) | p-value |
| --- | --- | --- | --- | --- |
| **Age, years** | 66.79 ± 9.91 | 68.48 ± 9.73 | 66.12 ± 9.91 | **<0.001** |
| **Diabetes mellitus** | 4,386 (33.5%) | 1,047 (28.0%) | 3,339 (35.7%) | **<0.001** |
| **Hypertension** | 6,111 (46.7%) | 1,700 (45.4%) | 4,411 (47.2%) | 0.067 |
| **Current smoking** | 3,040 (23.3%) | 770 (20.6%) | 2,270 (24.3%) | **<0.001** |
| **Opium use** | 2,162 (16.6%) | 610 (16.4%) | 1,552 (16.7%) | 0.618 |
| **FHpCVD** | 4,515 (34.5%) | 1,293 (34.5%) | 3,222 (34.4%) | 0.933 |
| **BMI, kg/m2** | 26.66 ± 3.78 | 26.06 ± 3.83 | 26.89 ± 3.74 | **<0.001** |
| **Previous_MI** | 4,745 (36.2%) | 1,294 (34.6%) | 3,451 (36.9%) | **0.012** |
| **Previous_HF** | 374 (2.9%) | 96 (2.6%) | 278 (3.0%) | 0.206 |
| **CKD** | 269 (2.1%) | 53 (1.4%) | 216 (2.3%) | **0.001** |
| **COPD** | 444 (3.4%) | 129 (3.5%) | 315 (3.4%) | 0.829 |
| **Previous CVA** | 865 (6.7%) | 240 (6.5%) | 625 (6.8%) | 0.575 |
| **PAD** | 238 (1.8%) | 71 (1.9%) | 167 (1.8%) | 0.670 |
| **Previous CABG** | 75 (0.6%) | 24 (0.6%) | 51 (0.5%) | 0.512 |
| **Previous PCI** | 1,011 (7.7%) | 292 (7.8%) | 719 (7.7%) | 0.832 |
| **LDL-C, mg/dL** | 93.66 ± 36.02 | 100.84 ± 38.78 | 90.79 ± 34.43 | **<0.001** |
| **Triglycerides, mg/dL** | 145.68 ± 80.40 | 123.64 ± 73.31 | 154.50 ± 81.41 | **<0.001** |
| **Creatinine, mg/dL** | 1.03 ± 0.56 | 1.01 ± 0.50 | 1.04 ± 0.58 | **0.007** |
| **EF, %** | 45.50 ± 9.24 | 46.24 ± 8.97 | 45.21 ± 9.32 | **<0.001** |
| **Number of grafts** |  |  |  | 0.233 |
| **1** | 217 (1.7%) | 60 (1.6%) | 157 (1.7%) |  |
| **2** | 1,385 (10.6%) | 424 (11.3%) | 961 (10.3%) |  |
| **3** | 4,854 (37.1%) | 1,349 (36.0%) | 3,505 (37.5%) |  |
| **4** | 5,339 (40.8%) | 1,553 (41.5%) | 3,786 (40.5%) |  |
| **5** | 1,294 (9.9%) | 358 (9.6%) | 936 (10.0%) |  |
| **OPCAB** | 1,236 (9.5%) | 329 (8.9%) | 907 (9.8%) | 0.104 |
| **ACEi** | 5,765 (45.4%) | 1,670 (46.3%) | 4,095 (45.1%) | 0.233 |
| **Statin** | 11,503 (89.8%) | 3,270 (89.7%) | 8,233 (89.8%) | 0.952 |
| **Aspirin** | 9,188 (72.4%) | 2,619 (72.6%) | 6,569 (72.4%) | 0.789 |
| **Beta Blockers** | 10,498 (81.6%) | 3,001 (82.0%) | 7,497 (81.4%) | 0.423 |

HDL-C, high density lipoprotein cholesterol; FHpCVD, family history of cardiovascular disease; BMI, body mass index; MI, myocardial infarction; HF, heart failure; CKD, chronic kidney disease; COPD, chronic obstructive pulmonary disease; CVA, cerebrovascular accident; PAD, peripheral arterial disease; CABG, coronary artery bypass grafting; PCI, percutaneous coronary intervention; LDL-C, low density lipoprotein cholesterol; EF, ejection fraction; OPCAB, off-pump coronary artery bypass; ACEi, Angiotensin-converting-enzyme inhibitors; ARB, Angiotensin receptor blockers.

**Supplemental Table 3.** One-year incidence rate of mortality and MACCE components following CABG

| **1-year Incidence rate** | **Estimate** | **Lower** | **Upper** | **No. of events** |
| --- | --- | --- | --- | --- |
| ACS in desirable HDL group | 23.40 | 19.04 | 28.46 | 100 |
| ACS in low HDL group | 28.70 | 25.83 | 31.81 | 364 |
| TIA/stroke in desirable HDL group | 5.85 | 3.78 | 8.64 | 25 |
| TIA/stroke in low HDL group | 8.59 | 7.06 | 10.36 | 109 |
| Mortality in desirable HDL group | 15.19 | 11.75 | 19.32 | 66 |
| Mortality in low HDL group | 17.55 | 15.35 | 19.99 | 227 |
| Revascularization in desirable HDL group | 0.23 | 0.00 | 1.30 | 1 |
| Revascularization in low HDL group | 0.55 | 0.22 | 1.13 | 7 |

ACS: Acute coronary syndrome, MACCE: Major adverse cardiovascular and cerebrovascular events,

TIA: Transient ischemic attack.

**Supplemental Figure 1.**  Survival curve for (A) 1-year post surgery mortality; and (B) MACCE outcomes (unadjusted) among male patients


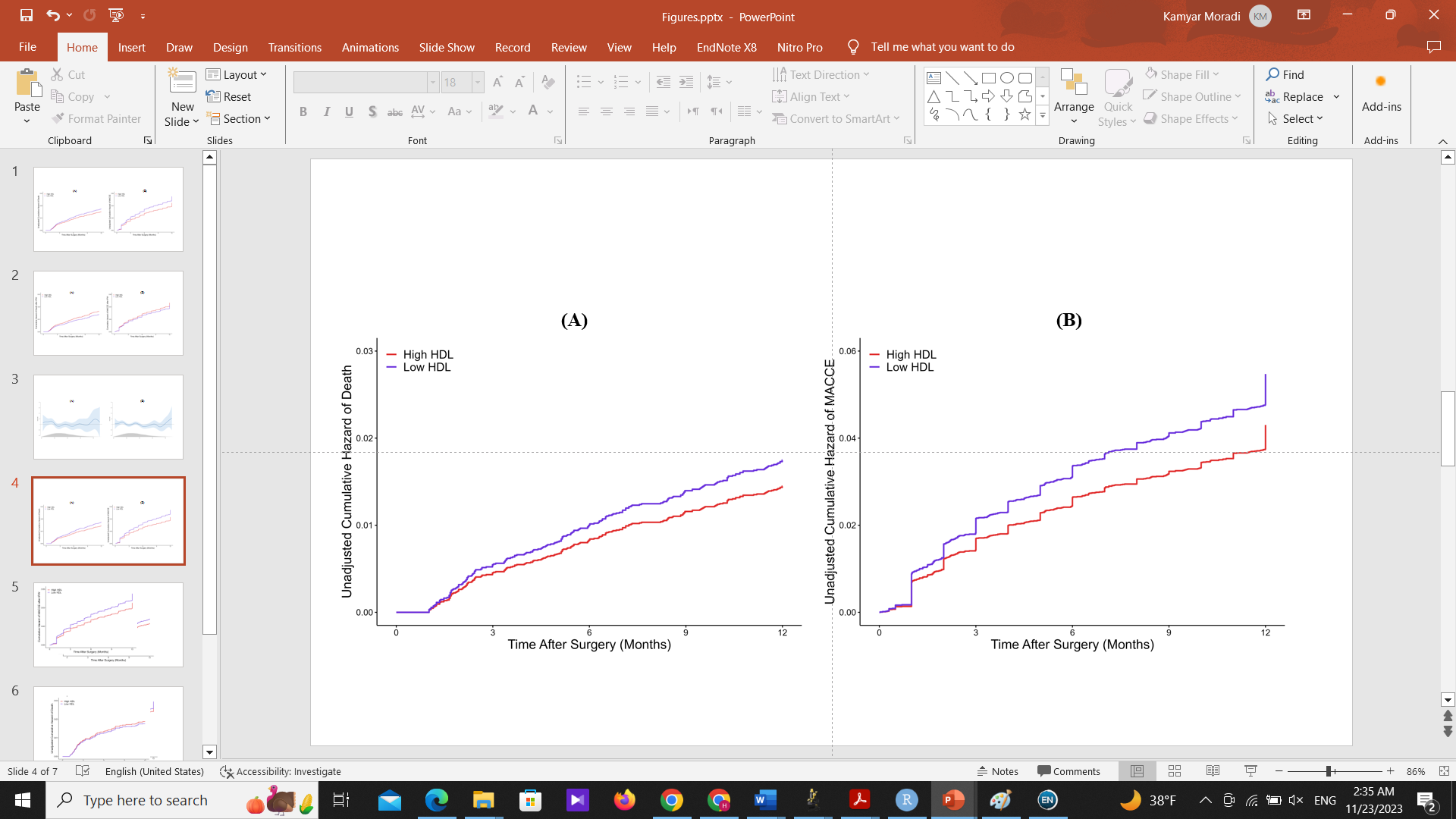


**Supplemental Figure 2.** Survival curve for (A) 1-year post surgery mortality; and (B) MACCE outcomes (adjusted) among male patients


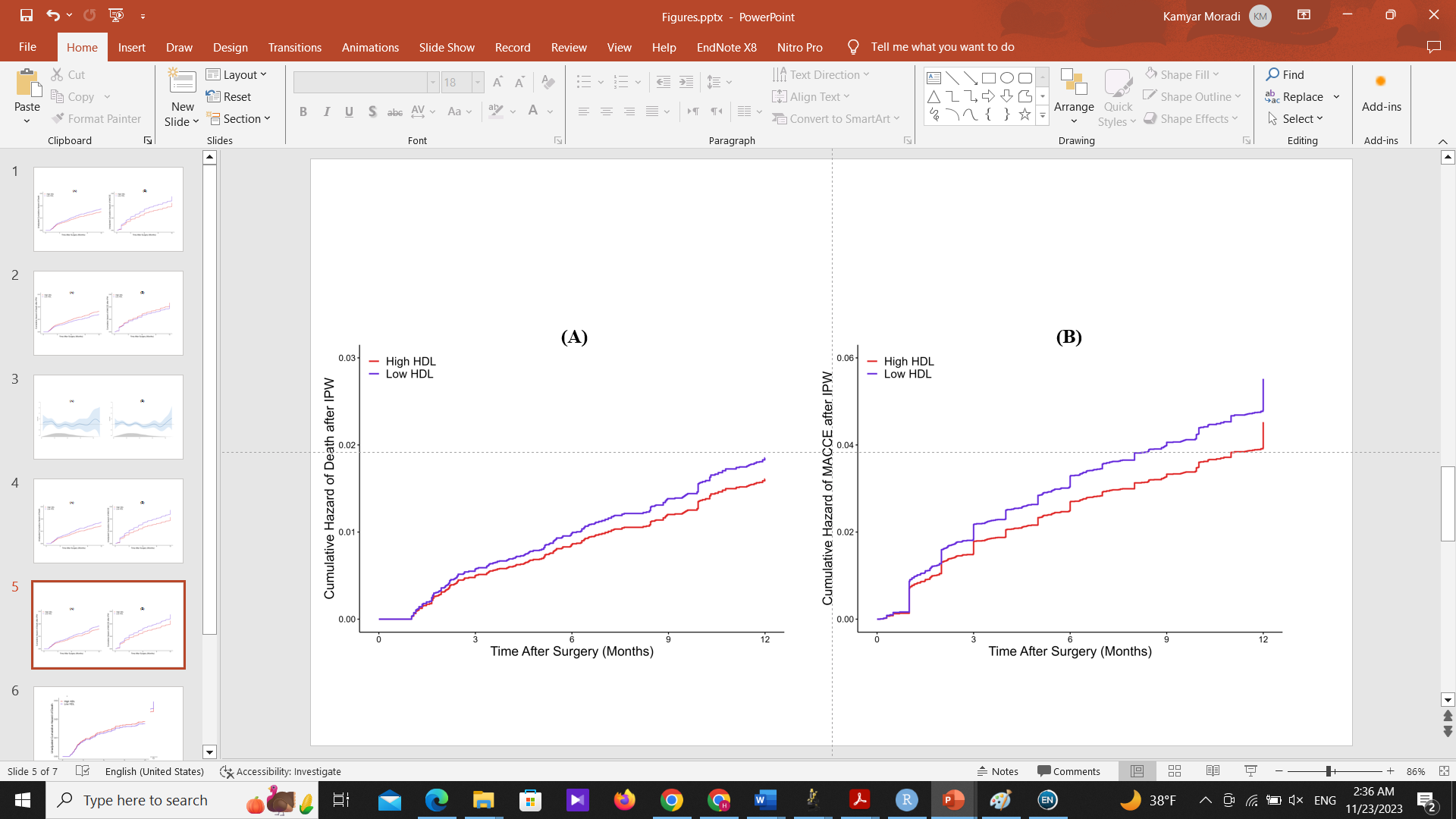


**Supplemental Figure 3.**  Survival curve for (A) 1-year post surgery mortality; and (B) MACCE outcomes (unadjusted) among female patients


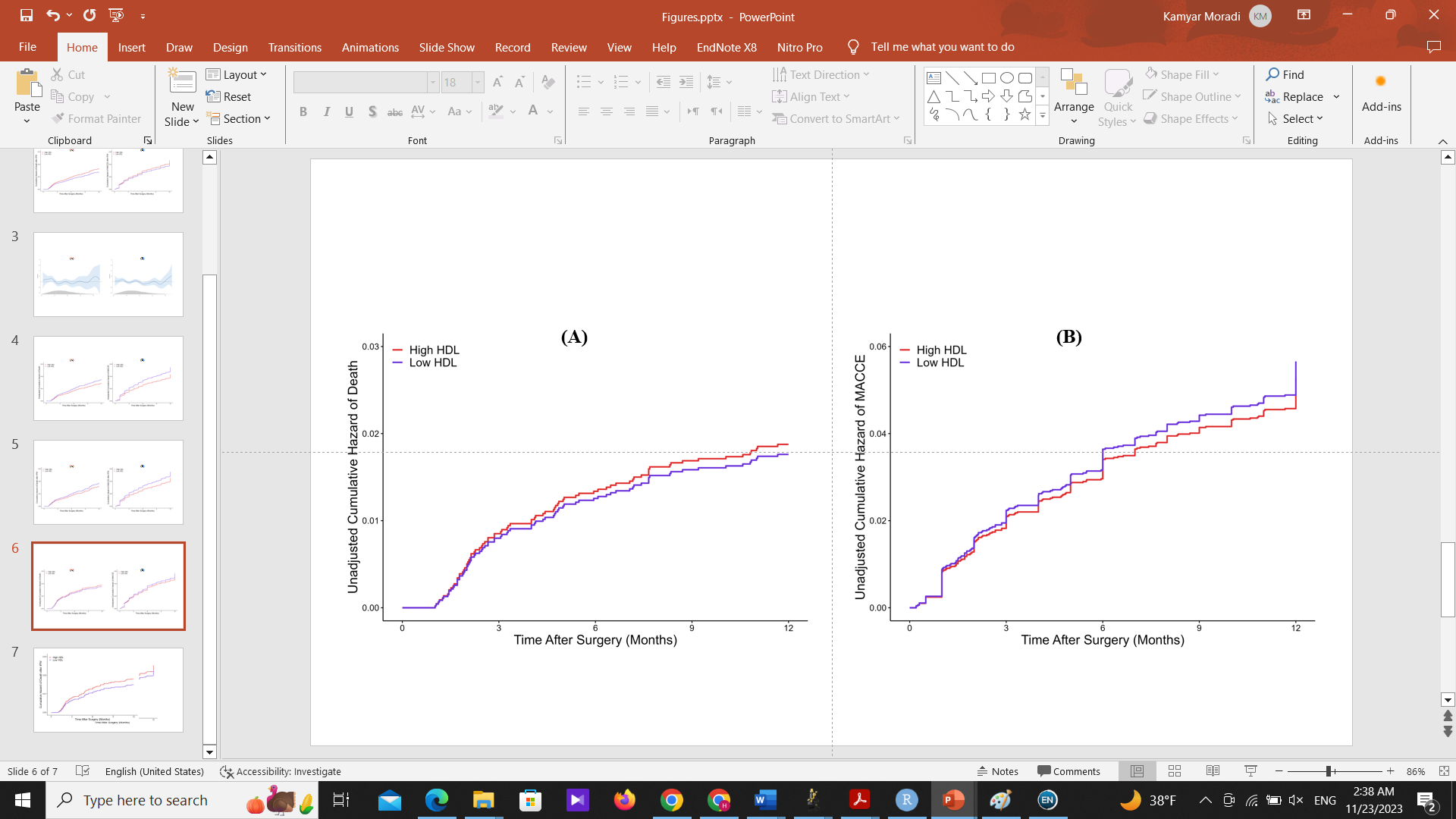


**Supplemental Figure 4.** Survival curve for (A) 1-year post surgery mortality; and (B) MACCE outcomes (adjusted) among female patients


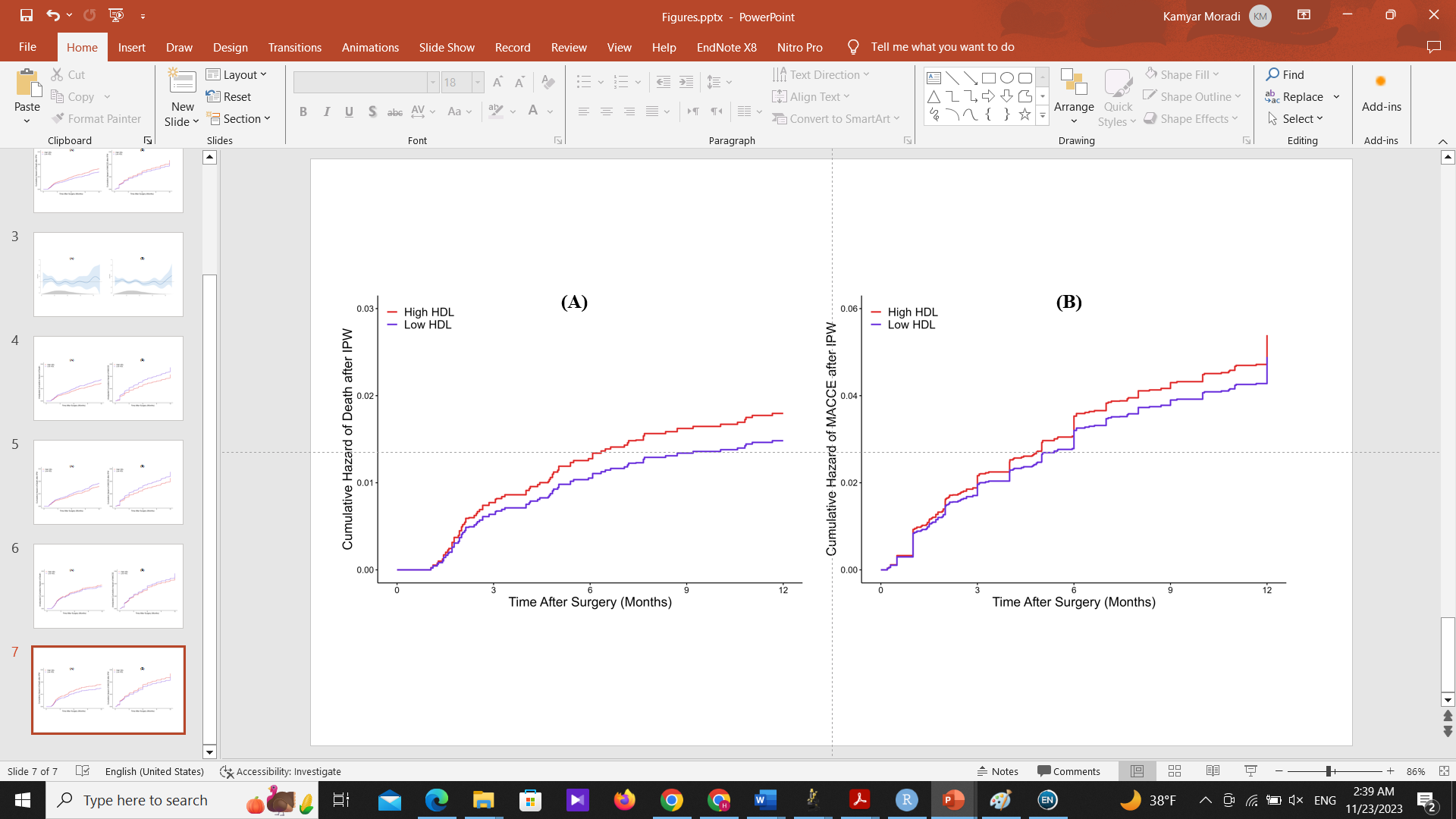


**Supplemental Table 4.** One-year post surgery outcomes in female subgroup

| **Outcomes** | **No. of events** | **Incidence rate per 1000 person-years (95% CI)** | **HR (95% CI)** | ***P* value** |
| --- | --- | --- | --- | --- |
| **Incident death (Crude)** | | | | |
| **Desirable HDL-C** | 13/706 | 18.83 (10.03-32.20) | Reference | 0.835 |
| **Low HDL-C** | 68/3968 | 17.69 (13.74-22.43) | 0.93 (0.51-1.69) |  |
| **Incident death (IPW-adjusted)** | | | | |
| **Desirable HDL-C** | 13/706 | 18.83 (10.03-32.20) | Reference | 0.643 |
| **Low HDL-C** | 68/3968 | 17.69 (13.74-22.43) | 0.82 (0.36-1.85) |  |
| **Incident MACCE (Crude)** | | | | |
| **Desirable HDL-C** | 36/706 | 53.16 (37.23-73.60) | Reference | 0.715 |
| **Low HDL-C** | 214/3968 | 56.75 (49.40-64.89) | 1.06 (0.75-1.52) |  |
| **Incident MACCE (IPW-adjusted)** | | | | |
| **Desirable HDL-C** | 34/706 | 53.16 (37.23-73.60) | Reference | 0.727 |
| **Low HDL-C** | 214/3968 | 56.75 (49.40-64.89) | 0.90 (0.52-1.56) |  |

HR, hazard ratio; CI, confidence interval; HDL-C, high density lipoprotein cholesterol; MACCE, major adverse cardiovascular and cerebrovascular events.

**Supplemental Table 5.** One-year post surgery outcomes in male subgroup

| **Outcomes** | **No. of events** | **Incidence rate per 1000 person-years (95% CI)** | **HR (95% CI)** | ***P* value** |
| --- | --- | --- | --- | --- |
| **Incident death (Crude)** | | | | |
| **Desirable HDL-C** | 53/3745 | 14.50 (10.86-18.97) | Reference | 0.237 |
| **Low HDL-C** | 159/9353 | 17.50 (14.89-20.44) | 1.20 (0.88-1.64) |  |
| **Incident death (IPW-adjusted)** | | | | |
| **Desirable HDL-C** | 53/3745 | 14.50 (10.86-18.97) | Reference | 0.573 |
| **Low HDL-C** | 159/9353 | 17.50 (14.89-20.44) | 1.15 (0.70-1.87) |  |
| **Incident MACCE (Crude)** | | | | |
| **Desirable HDL-C** | 155/3745 | 43.11 (36.59-50.46) | Reference | 0.009 |
| **Low HDL-C** | 490/9353 | 54.99 (50.22-60.08) | 1.27 (1.06-1.52) |  |
| **Incident MACCE (IPW-adjusted)** | | | | |
| **Desirable HDL-C** | 155/3745 | 43.11 (36.59-50.46) | Reference | 0.109 |
| **Low HDL-C** | 490/9353 | 54.99 (50.22-60.08) | 1.22 (0.95-1.55) |  |

HR, hazard ratio; CI, confidence interval; HDL-C, high density lipoprotein cholesterol; MACCE, major adverse cardiovascular and cerebrovascular events.
